# Supplementary material for: The Regulatory Network of Sweet Corn (Zea mays L.) Seedlings under Heat Stress Revealed by Transcriptome and Metabolome Analysis
Source: Int J Mol Sci. 2023 Jun 29;24(13):10845. doi: 10.3390/ijms241310845 (PMC10341527; doi:10.3390/ijms241310845)
Supplement: Supplementary file 1 [file ijms-24-10845-s001.zip › Supplement Figures S1-S7.pdf]

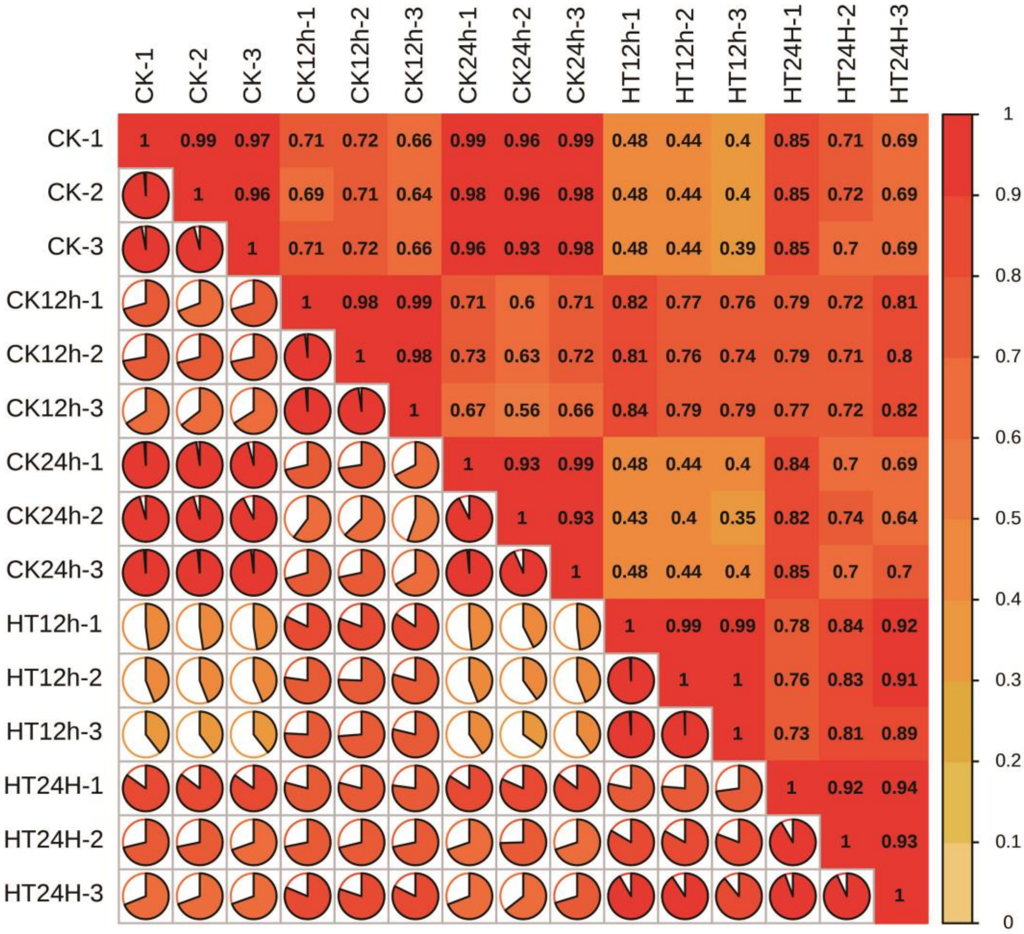

Figure S1. Correlation coefficient values for all the samples.

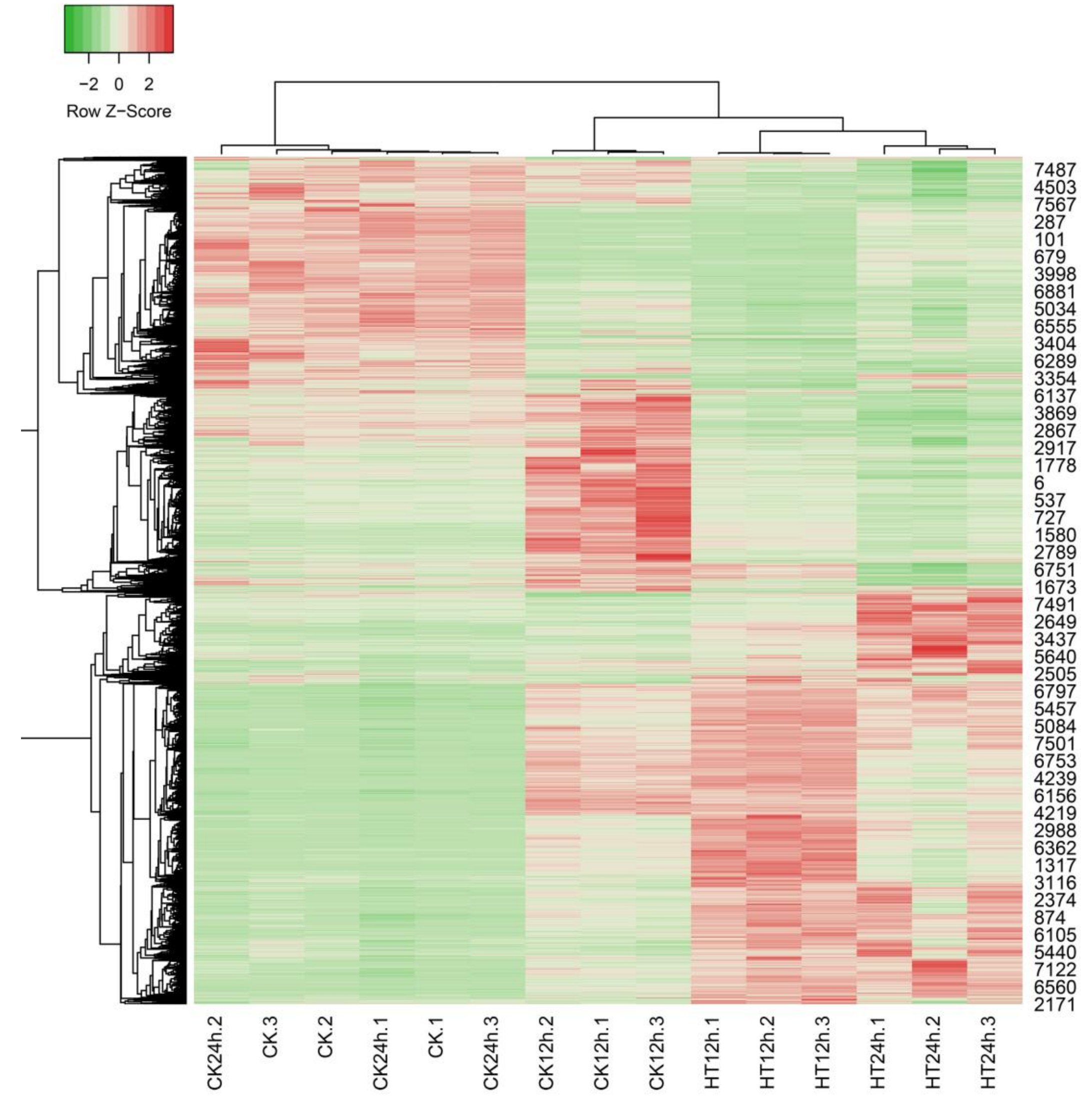

Figure S2. Heatmap for the DEGs of all the samples between different time points after heat stress.

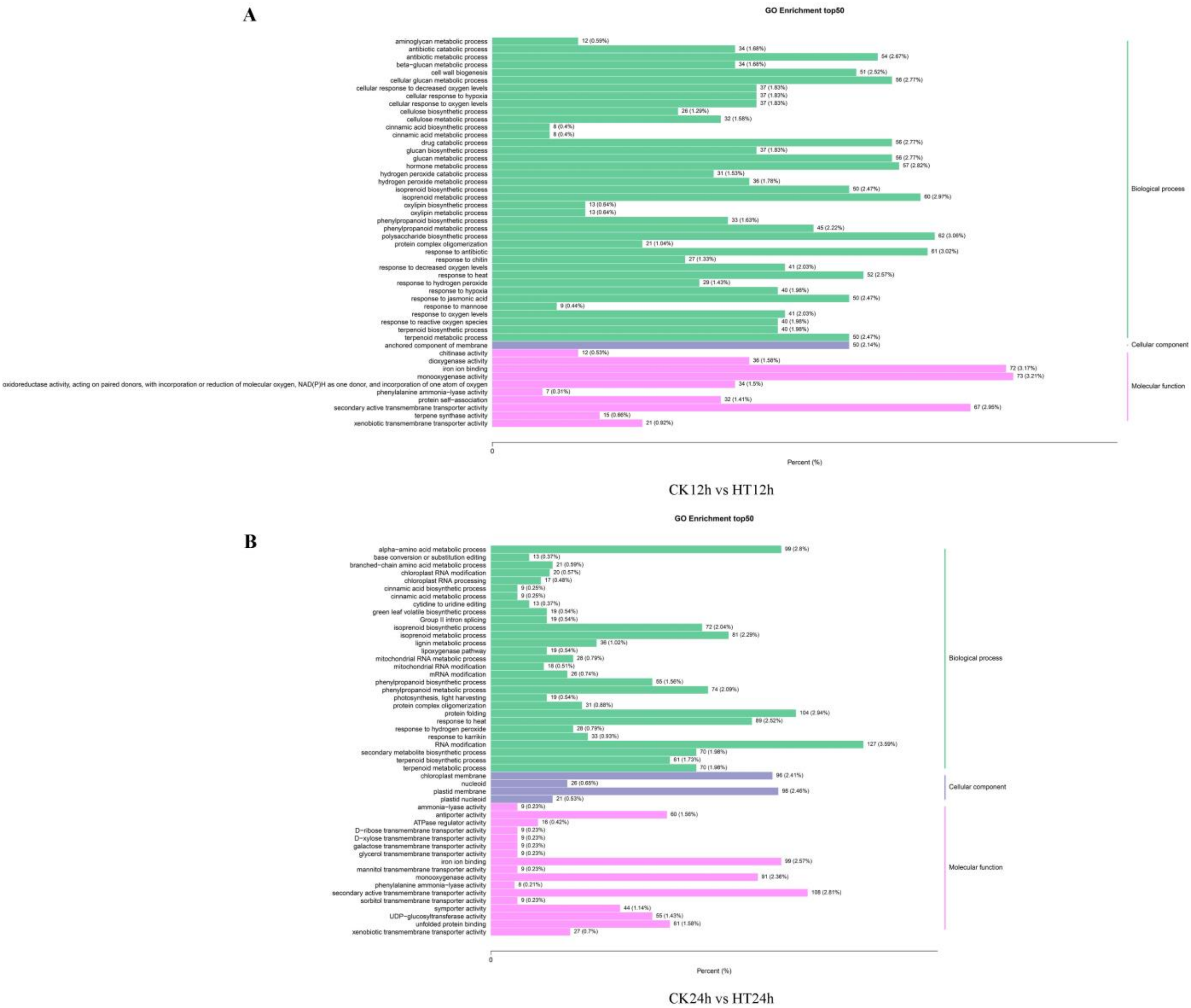

Figure S3. Top 50 items for GO enrichment for CK12h vs. HT 12h (A) and CK24h vs. HT 24h (B). Different colors of the bars in A, B stand for Biological process (green), Cellular component (purple), and Molecular function (pink).

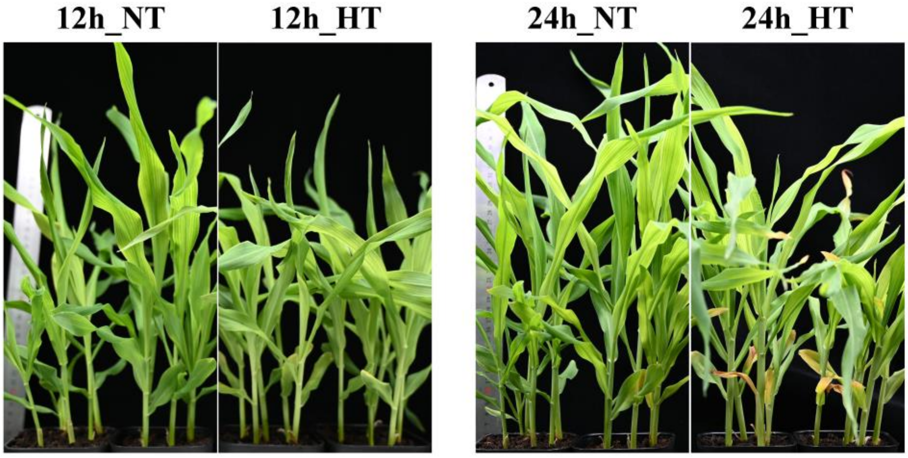

Figure S4. Images for sweet corn with or without heat stress after 12 h and 24 h. “NT” means “no treatment”, “HT” means “heat treatment”.

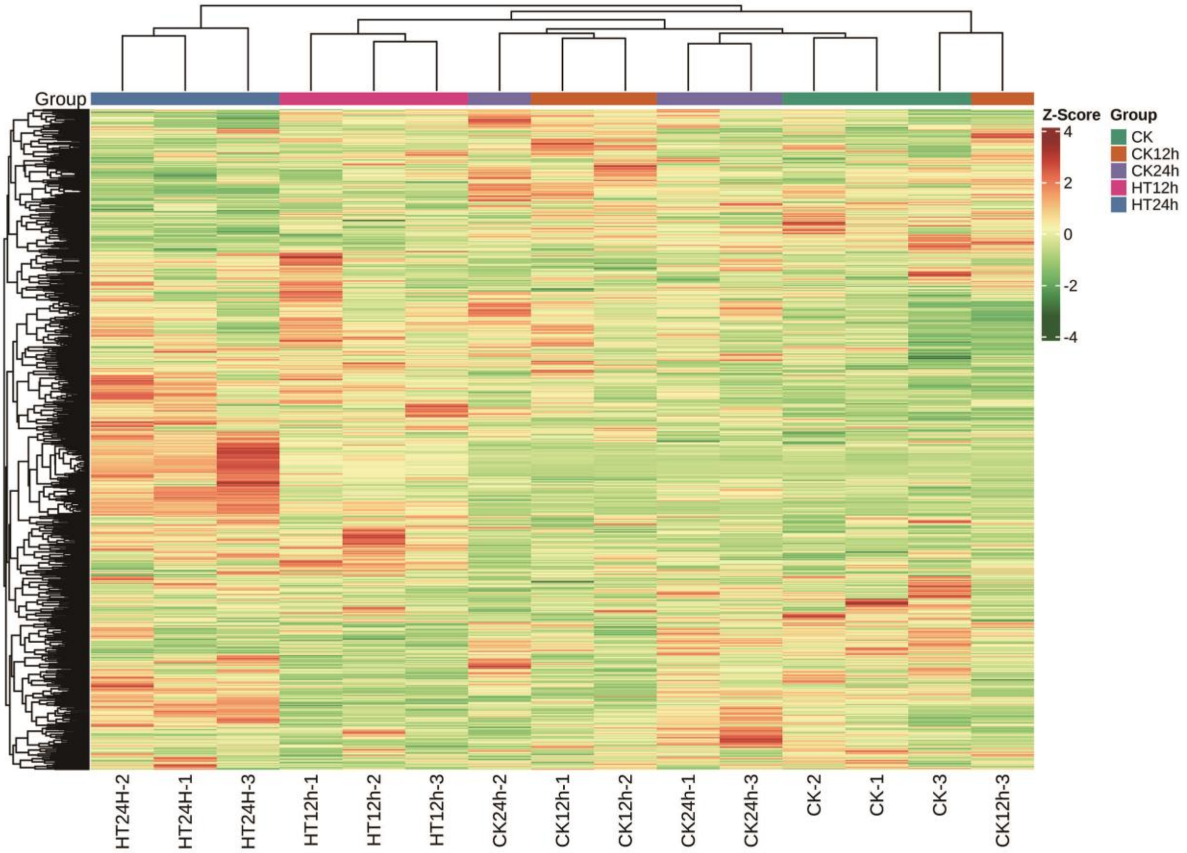

Figure S5. Heatmap of all the metabolites detected after heat stress.

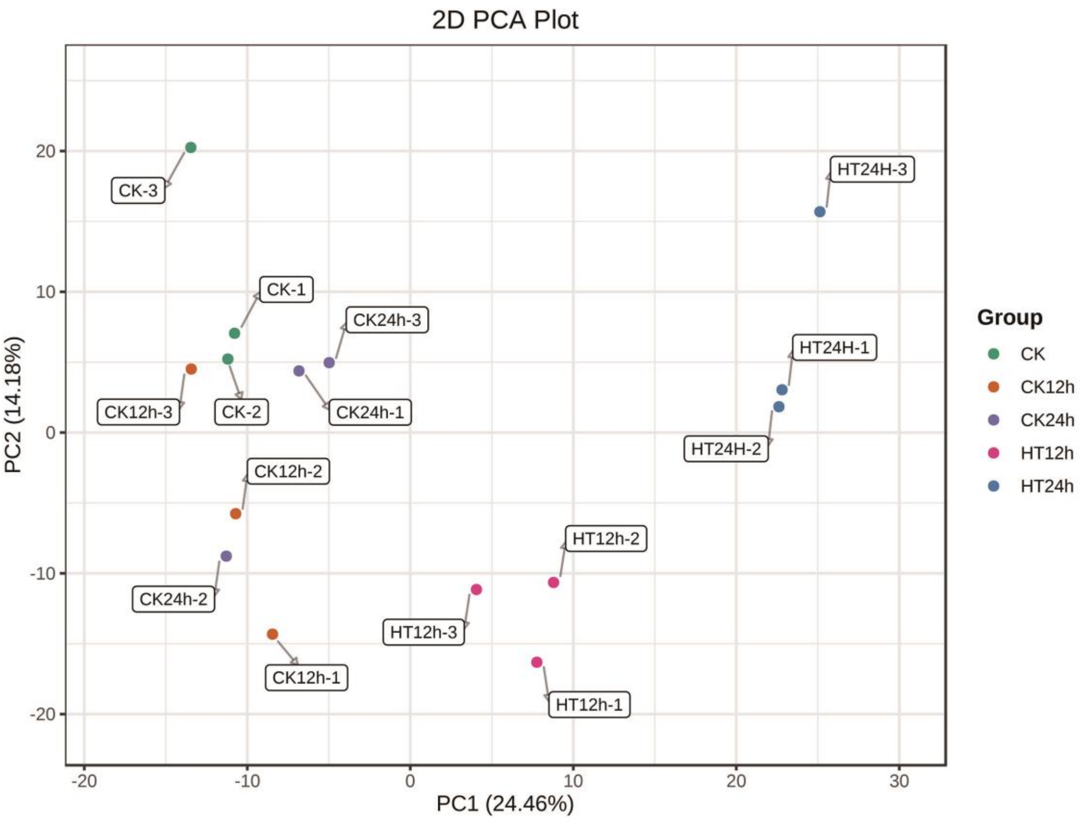

Figure S6. PCA analysis for metabolites detected after heat stress.

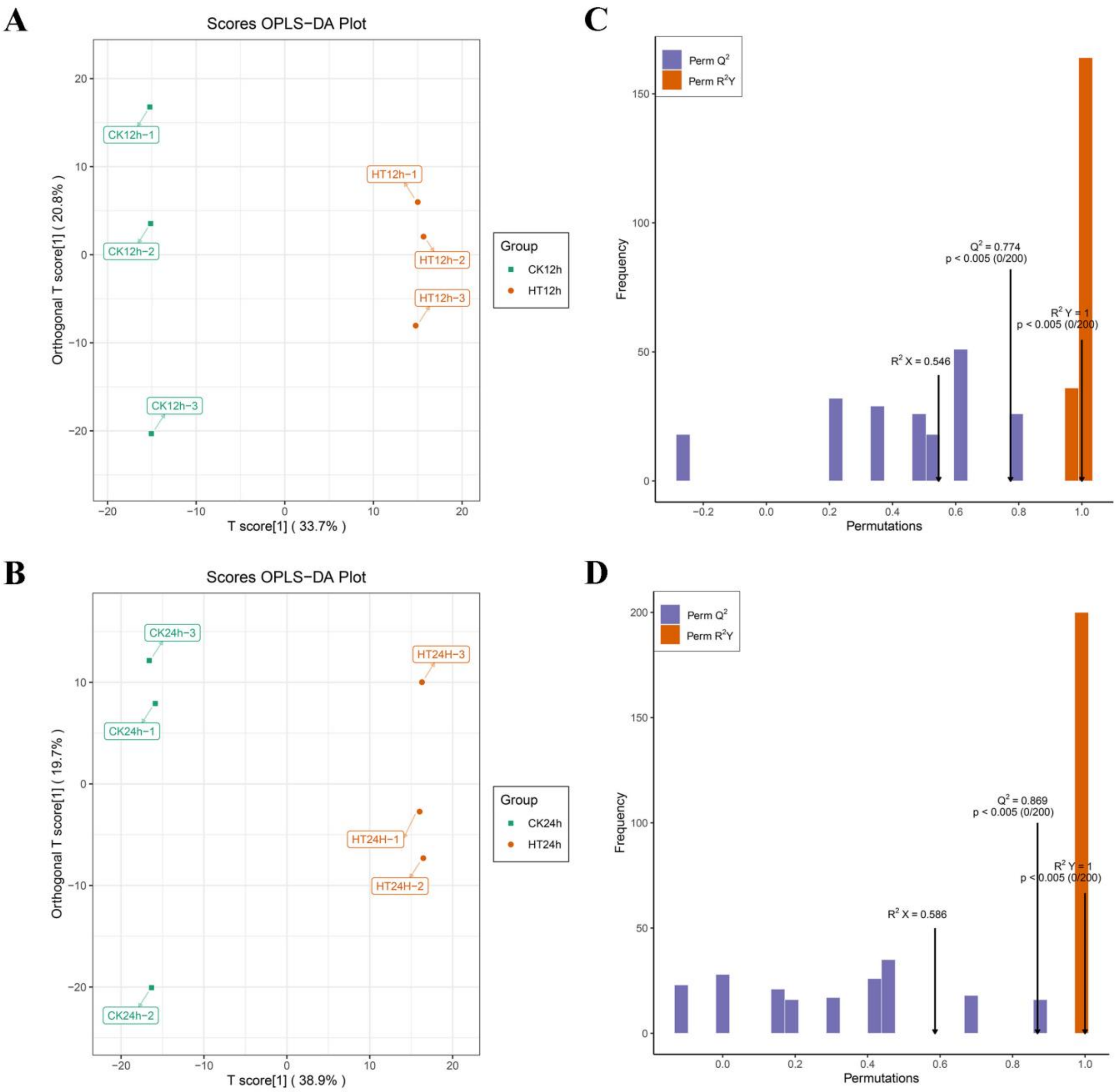

Figure S7. OPLS-DA scores and permutation maps. OPLS-DA score maps for CK after 12h vs. HT after 12h (A) and CK after 24h vs. HT after 24h (B). OPLS-DA permutation maps for CK after 12h vs. HT after 12h (C) and CK after 24h vs. HT after 24h (D).
